# A Passive Wireless Crack Sensor Based on Patch Antenna with Overlapping Sub-Patch

**DOI:** 10.3390/s19194327

**Published:** 2019-10-07

**Authors:** Songtao Xue, Zhuoran Yi, Liyu Xie, Guochun Wan, Tao Ding

**Affiliations:** 1 Department of Disaster Mitigation for Structures, Tongji University, Shanghai 200092, China; xue@tongji.edu.cn (S.X.); yzr1997@tongji.edu.cn (Z.Y.); 2 Department of Architecture, Tohoku Institute of Technology, Sendai 982-8577, Japan; 3 Key Laboratory of Performance Evolution and Control for Engineering Structures of Ministry of Education, Tongji University, Shanghai 200092, China; 4 Department of Electronic Science and Technology, Tongji University, Shanghai 200092, China; wanguochun@tongji.edu.cn; 5 Institute of Precision Optical Engineering, School of Physics Science and Engineering, Tongji University, Shanghai 200092, China; dingtao@tongji.edu.cn

**Keywords:** passive wireless sensor, patch antenna, crack width monitoring, covered radiation patch

## Abstract

Monolithic patch antennas for deformation measurements are designed to be stressed. To avoid the issues of incomplete strain transfer ratio and insufficient bonding strength of stressed antennas, this paper presents a passive wireless crack sensor based on an unstressed patch antenna. The rectangular radiation patch of the proposed sensor is partially covered by a radiation sub-patch, and the overlapped length between them will induce the resonate frequency shift representing the crack width. First, the cavity model theory is adopted to show how the resonant frequencies of the crack sensor are related to the overlapped length between the patch antenna and the sub-patch. This phenomenon is further verified by numerical simulation using the Ansoft high-frequency structure simulator (HFSS), and results show a sensitivity of 120.24 MHz/mm on average within an effective measuring range of 1.5 mm. One prototype of proposed sensor was fabricated. The experiments validated that the resonant frequency shifts are linearly proportional to the applied crack width, and the resolution is suitable for crack width measuring.

## 1. Introduction 

Nowadays, bridges, civil architectures, and mechanical engineering systems continue to provide service despite aging and associated damage accumulation [1]. Failure of these structures can become a serious threat, causing both life and property loss [2]. Furthermore, to a great extent this type of structural failure can be due to the formation and expansion of cracks [3]. Hence, crack monitoring of aged and damaged structures is progressively becoming more important. 

Crack monitoring sensors are categorized as wired or wireless types. Cables for wired crack sensors supply energy power and transmit data. For its individual functionality, a crack’s existence and growth can be directly monitored by crack sensors, such as the optical fiber grating sensor [4,5], capacitance-based sensor [6], conductive area sensor [7], resistance based sensor [8], infrared sensor [9], and optical sensor [10,11]. These cable-powered sensors usually show reliable accuracy and high stability. However, the installation and application of wired crack sensors is both time- and labor-consuming due to the cable deployment and periodical manual inspection. Wireless crack sensors can significantly reduce instrumentation time and system cost [12,13]. However, wireless sensing devices usually operate on external power sources, such as the photovoltaic (PV) cell [14], thermoelectric generator (TEG) [15] and piezoelectric (PE) energy harvesting [16]. All of these power sources are expensive and prone to fail during long-term service [17].

In recent years, several passive wireless sensing techniques have been proposed to avoid cables These techniques and their accompanying devices have approached crack sensing in different ways, such as surface acoustic wave (SAW)-based systems [18], inductive coupled systems [19], radio-frequency identification (RFID) enabled systems [20,21], and antenna based chipless sensing systems [22,23]. With the adoption of passive wireless sensing technologies, costs have been reduced, and sensors have become ubiquitous. Among them, patch- antenna-based sensors have the advantage of simple configuration, multimodality, and other merits [24,25]. Yi et al. [13] proposed a patch antenna-based crack sensing system by attaching a monolithic patch antenna on the surface of structures and analyzing the resonant frequency shift of the antenna. Lange et al. [26] proposed a qualitative crack sensing method for concrete structures by embedding a patch antenna inside the concrete. The attenuation of the electromagnetic wave through the concrete leading to its patch antenna depends on the concrete’s aggregate distribution, which is affected by the cracks inside the concrete. Mohammad et al. [27,28] detected crack orientation by analyzing the crack-induced shifts of two fundamental resonant frequencies. The crack orientation can be sensed by the direction of the resonant frequency shift, while the value of the shift is affected by the width of the crack. Marindra et al. [29] also proposed a patch antenna-based crack sensor applied to crack width sensing and crack orientation sensing. Four dipole resonators are attached on each patch antenna as labels to achieve distributed sensing. These patch-antenna-based sensors generally utilize the resonant frequency shift of a one-piece antenna when the antenna is stretched or torn open.

However, for the sensors using monolithic patch antennas, the issues of incomplete strain transfer ratio, insufficient bonding strength, and randomness of crack propagation will compromise the sensor sensitivity and complicate the calibration process [30]. These issues will pose obstacles to the mass production of the one-piece patch antenna sensors. To overcome these difficulties, unstressed antennas are the alternative solution for sensing the displacement or crack width. Caizzone et al. [31] proposed a crack sensor made of two mutual coupling planar-inverted F antennas, which used coupled antennas’ phase changing to show the change in the distance between these two antennas. The crack width, when measured as the width of the crack, is nearly equal to the distance variation between two antennas. However, the measured phase is affected by the interrogation distance between the coupled antennas and reader, which means the reader position must be fixed according to monitoring process. Xue et al. [32] designed a passive displacement sensor based on a normal mode helical antenna using the resonant frequency shift induced by the relative movement between a helical antenna and an inserted dielectric rod. Xue et al. [30] also presented a crack sensor based on a patch antenna, which is fed by a pair of microstrip lines forming a parallel plate capacitor. The crack width can be sensed by measuring the relative movement between two microstrip lines without any stress in the patch antenna. The manufactured sensors were tested in a cabled environment with a fine resolution of 0.01 mm, and the antenna-based crack sensor can design to be a wireless type by change the excitation source. However, it is currently impractical to test chipped crack sensors via wireless interrogation because a commercial RFID chip over 2.4 GHz is not available in the market. 

Previous studies of Xue et al. [30] have utilized a parallel plate capacitor formed by two microstrip lines as a sensing unit. This paper presents another alternative solution to unstressed antennas, wherein the electric length of the combined radiation patch is correlated with the crack width to simplify the antenna design. Thus, the authors propose the development of a crack sensor based on a rectangular patch antenna combined with a movable radiation patch. The total length of the combined radiation patch would be altered by the relative movement between the patch antenna and the dielectric board, leading to a shift of resonant frequency in the sensing system. This chipless crack sensor can be interrogated wirelessly using a wide-band patch antenna, which can serve as both the transmitter and receiver.

This paper is organized as follows. Section 2 introduces the concept of a concrete crack sensor based on a patch antenna with a covered radiation patch and illustrates the sensing mechanism using the cavity model theory. This section introduces the innovative concept of one prototype of the crack sensor. In Section 3, the appropriate dimension parameters are determined for the sensing system. Section 4 describes the fabrication of sensors and the instrumentation setup of experiments. The resonant frequency of the chipless crack sensor is analyzed wirelessly by a wide band antenna over the sensor. Conclusions are then drawn, and future research potential is discussed.

## 2. Crack Sensor Using a Combined Radiation Patch

Monolithic patch antennas as strain or crack sensing units are designed to be stretched. The alternative way is to make the sensing unit unstressed by combining two radiation patches, whose total electric length depends on the overlapped length of two components. 

**Figure 1:**
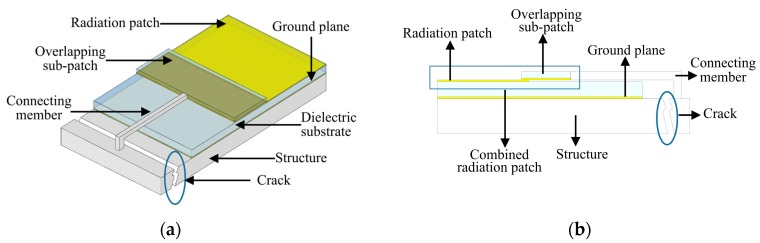
Concept of crack sensor using a patch antenna as shown by: (**a**) an axonometric drawing; (**b**) a front view.

According to the cavity model theory, a patch antenna composed of a radiation patch and a ground plane can be treated as a resonant cavity, and the resonant frequencies of the cavity are related to the dimension of the radiation patch [33]. Based on this principle, a patch-antenna-based crack sensor is proposed, as illustrated in Figure 1. This crack sensor consists of a partially-covered patch antenna and an overlapping sub-patch with the same width as the patch antenna. The overlapping sub-patch is placed over the underlying rectangular radiation patch, and the electric current induced by interrogation waves can flow within the combined radiation patch. For practical use, the patch antenna is placed on one side of the crack, while the sub-patch is connected with the other side of the crack. Once the crack is expanded, the relative position between the patch antenna and the movable sub-patch will change and lead to an increased electric length of the combined radiation patch, which produces the crack sensor’s shift in resonant frequencies. 

### 2.1. Theoretical Calculation for Proposed Crack Sensor

**Figure 2:**
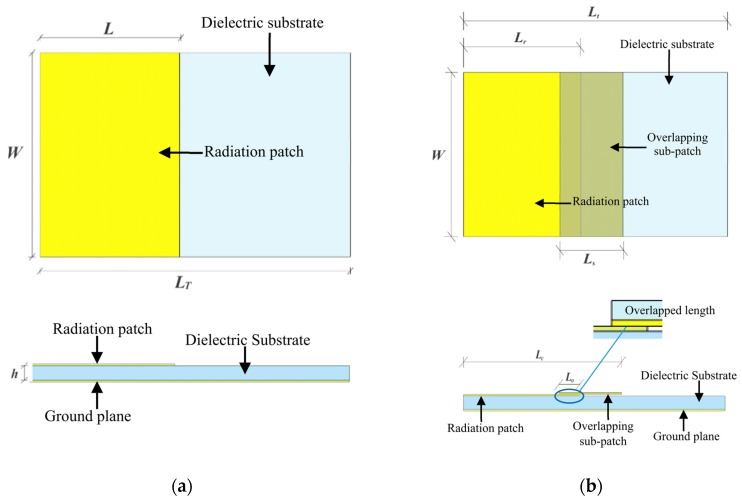
A normal rectangular patch antenna and proposed crack sensor. (**a**) A normal rectangular patch antenna (**b**) The proposed crack sensor using sub-patch.

For a normal rectangular patch antenna [33] as shown in Figure 2a, assuming the electrical length is $L_{e}$ and the electrical width is $W_{e}$, the resonant frequencies can be calculated as [33]:

(1) $$f_{mn}=\frac{c}{2\pi \sqrt{\varepsilon }}\sqrt{{\left(\frac{m\pi }{L_{e}}\right)}^{2}+{\left(\frac{n\pi }{W_{e}}\right)}^{2}},$$

where m and n are the orders in longitudinal direction and transverse direction respectively, c is the speed of light, ε is the dielectric constant of the dielectric substrate, $f_{mn}$ is the resonant frequency when the antenna is resonant at m orders in longitudinal direction and n orders in transverse direction.

Because the longitudinal elongation only affects the length of the radiation patch, the fundamental resonant frequency in longitudinal direction can be calculated as

(2) $$f_{10}=\frac{c}{2L_{e}\sqrt{\varepsilon }},$$

where $f_{10}$ is the fundamental resonant frequency in the longitudinal direction. The electric length can be calculated from the geometric dimensions of the radiation patch and the fringe extensions as

(3) $$L_{e}=L+\Delta L,$$

where L is the length of the radiation patch and ΔL is the line extension. When the substrate height h is much smaller than the dimensions of the radiation patch, the line extension ΔL can be neglected [33]. Therefore, Equation (3) can be reduced to

(4) $$f_{10}=\frac{c}{2L\sqrt{\varepsilon }}$$

Analogously, applying Equation (4) to the proposed crack sensor with combined radiation patches as shown in Figure 2b, the fundamental resonant frequency can be calculated as:

(5) $$f_{10c}=\frac{c}{2(L_{s}+L_{r}-L_{o})\sqrt{\varepsilon_{e}}}.$$

where $f_{10c}$ is the fundamental resonant frequency in the longitudinal direction for the proposed crack sensor, $L_{r}$ is the length of the radiation patch, $L_{s}$ is the length of the sub-patch, and $L_{o}$ is the overlapped length between the radiation patch and the covering sub-patch.

When a crack opens wider, the overlapped length would decrease, which changes the resonant frequency as:

(6) $$f_{10c}^{\prime }=\frac{c}{2(L_{s}+L_{r}-L_{o}-\Delta L_{o})\sqrt{\varepsilon_{e}}},$$

(7) $$\Delta L_{o}=d,$$

where $\Delta L_{o}$ is the difference of the overlapped length after the crack opens and is equal to the crack width d. We can then rewrite Equation (6) as:

(8) $$f_{10c}^{\prime }=\frac{c(L_{s}+L_{r}-L_{o}-\Delta L_{o})}{2{\left(L_{s}+L_{r}-L_{o}-\Delta L_{o}\right)}^{2}\sqrt{\varepsilon_{e}}}.$$

As the crack width d is trivial compared with the dimensions of the crack sensor, the increment in denominator can be ignored and Equation (8) can be rewritten as:

(9) $$f_{10c}^{\prime }=\frac{c(L_{c}-\Delta L_{o})}{2L_{c}{}^{2}\sqrt{\varepsilon_{e}}},$$

where $L_{c}$ is the combined length of the crack sensor, which can be calculated as:

(10) $$L_{c}=L_{s}+L_{r}-L_{o}.$$

The shift of the fundamental resonant frequency in longitudinal direction can be then determined by Equation (11):

(11) $$\Delta f_{10c}=\frac{\Delta L_{o}}{2L_{c}{}^{2}\sqrt{\varepsilon_{e}}}.$$

where $\Delta f_{10c}$ is the shift of the fundamental resonant frequency in the longitudinal direction; that is, the fundamental resonant frequency in the longitudinal direction changes linearly with the increasing overlapped length.

### 2.2. Design of Crack Sensors 

According to the theoretical calculation in Section 2.1, the sub-patch should contact fully with the radiation patch, which depends on the flatness of the surface between the sub-patch and the radiation patch. To achieve this, a prototype with a dielectric board over the sub-patch was fabricated to stiffen the patch, and the dimensions of the sensor are shown in Figure 2b. Other solutions could also be practical, such as enlarging the height of the sub-patch or finding a material of the sub-patch with higher stiffness.

This paper’s featured patch antenna is demonstrated as working at around 2.4 GHz in the laboratory’s available testing environment. The relationship between the fundamental resonant frequency in longitudinal direction and the dimension of the crack sensors is described in Equation (4), and the length of the combined radiation patch $L_{c}$ is calculated by Equation (10). 

The width of the patch antenna is defined as follows to ensure the radiation efficiencies [33]:

(12) $$W\leq \frac{c}{2f}\sqrt{\frac{\varepsilon_{e}+1}{2}},$$

where f is the working resonant frequency of the patch antenna.

Based on Equations (10) and (11), the setting domain for the crack sensor is determined as shown in Table 1.

To ensure the working performance of the sensors, an Ansoft high frequency structure simulator (HFSS) was used to simulate the crack sensors with different sizes, to be described in Section 3.

## 3. Modeling and Simulation

**Figure 3:**
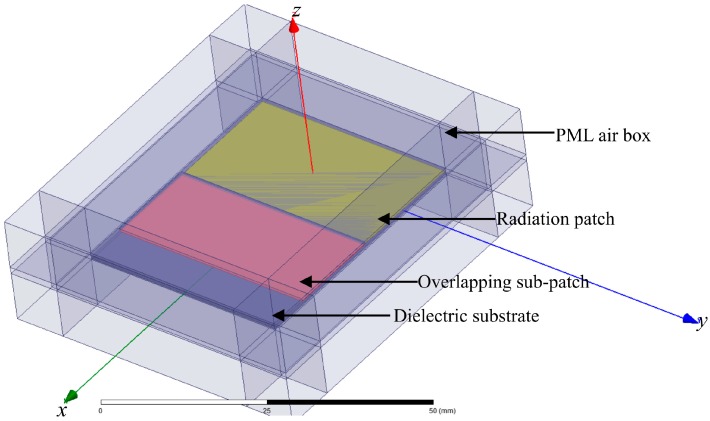
Schematic diagram of the crack sensor.

The radiation properties of the crack sensor are simulated using the Ansoft HFSS. Figure 3 shows the HFSS model, which consists of a patch antenna and a sub-patch. The sub-patch is made up of a copper sheet and a coated dielectric board. The material of the dielectric board is Rogers RT/®duroid 5880. Copper is the chosen material of the radiation patch. The crack sensor is arranged inside a perfectly matched layer (PML) air box with a radius of about a quarter wavelength to ensure computational accuracy of the far field radiation. The whole system is fed by a plane wave above the patch antenna. Because the electric field should be perfectly perpendicular to the surface, a perfect E was selected as the boundary condition of the ground plane. 

After calculating the fundamental resonant frequency within the setting range in Table 1 by HFSS, one set of the crack sensors with a better performance is proposed. The parameters are shown in Table 2.

**Figure 4:**
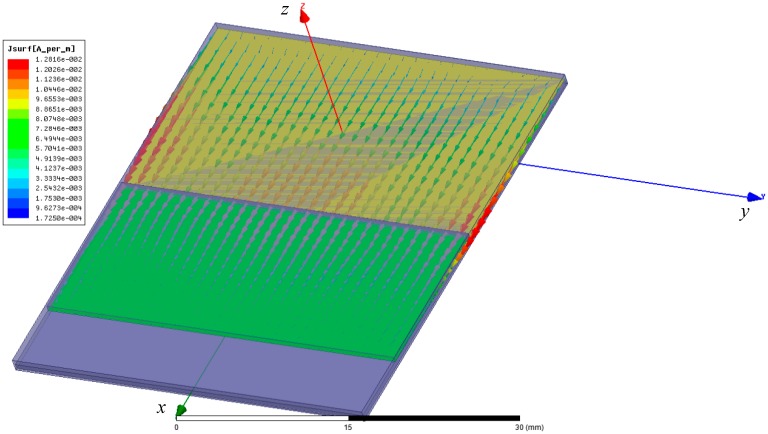
The induced current distribution pattern on the combined radiation patch for the fundamental mode.

Then, the simulation of the crack sensor with the moving sub-patch is carried out. In the initial state, the overlapped length between the radiation patch and sub-patch is 0 mm. With the movement of the sub-patch, the overlapped area expanded continuously until it reached 6 mm. The induced current distribution pattern on the combined radiation patch for the fundamental mode is shown in Figure 4. The current flows from the radiation patch to the movable sub-patch.

**Figure 5:**
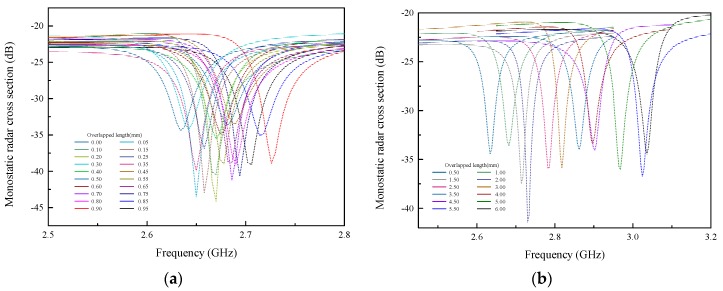
The monostatic radar cross section (RCS) of the crack sensor in a wide range: (**a**) 0 mm to 0.95 mm with a resolation of 0.05 mm; (**b**) 0.50 mm to 6.00 mm with a resolation of 0.5 mm.

**Figure 6:**
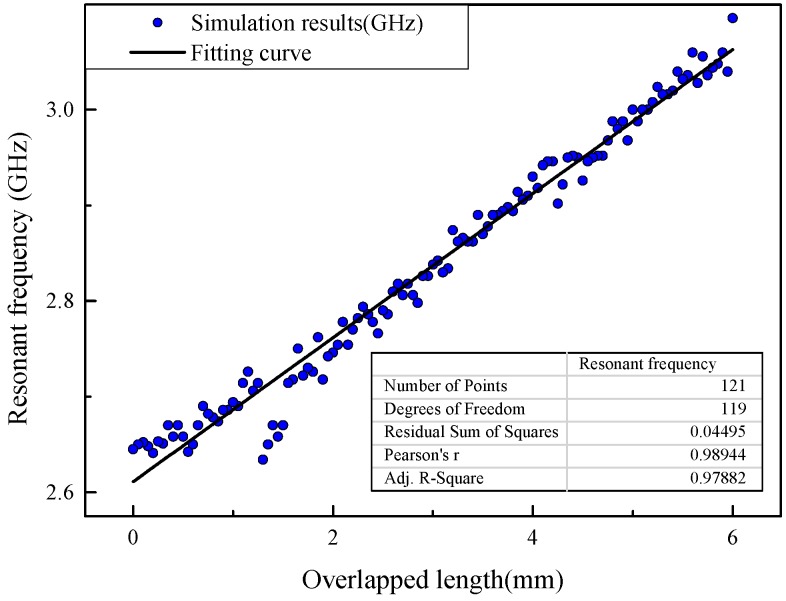
Relationship between fundamental resonant frequency in longitudinal direction and overlapped length.

One step of the movement is set at 0.05 mm. The monostatic radar cross section (RCS) around the fundamental resonant frequency in the longitudinal direction is acquired for each moving step, as shown in Figure 5. The fundamental resonant frequency of the displacement sensor is then extracted from each of the radar cross section (RCS) curves. The scatter diagram of the fundamental resonant frequency and overlapped length (which represents the crack width) is plotted in Figure 6. 

In general, a nearly linear relationship between the overlapped length and resonant frequency is observed in Figure 6, as described by Equation (10). The correlation coefficient of the fitted line is 0.9788, which shows the resonant frequency to nearly shift linearly with the increasing of overlapped length. The slope of the fitted line representing the sensitivity coefficient of the crack sensor is 0.07 GHz/mm. At the radiation boundary, the interrogating method and environmental situation in simulation are different than that found in the actual occasion. Experiments were designed and the procedures are defined in Section 4 to verify the performance of the proposed sensor.

## 4. Experiment

For the fabricated sensors, copper was selected as the material for the radiation patches. Rogers RT/duroid 5880 was selected as the middle substrate of the rectangular patch antenna and the substrate of sub-patch. The patch antenna was produced in laboratory and the fundamental resonant frequency of the crack sensor was interrogated wirelessly by a wide-band antenna.

### 4.1. Antenna Production in Laboratory

The antenna used in this study’s experiments was produced in the authors’ laboratory. A sandwich of a selected length and width having a dielectric substrate and two copper sheets coated on the both side of the dielectric substrate was used for antenna making. First of all, a heat toner transfer paper was cut to match the same width and length as the radiation patch. The paper’s surface was then covered with toner by laser printer. The paper was then added as a sandwiched layer of the radiation patch. Another sheet of paper with the same dimensions as that of the ground plane was disposed and then attached to the other copper sheet of the sandwich. A thermal transfer printer was used to transfer the toner from the paper to the copper sheet of the sandwich. The sandwich was put into the thermal transfer printer ten times to concern the toner thar was attached to the copper sheets. The heat toner transfer paper was then casted off. Finally, the sandwich coated with tonner was placed into a kind of corrosive liquid. The uncoated area in the sandwich was corroded, and the sandwich after corrosion served as the patch antenna used in this experiment. The sub-patch was produced in the same way. 

**Figure 7:**
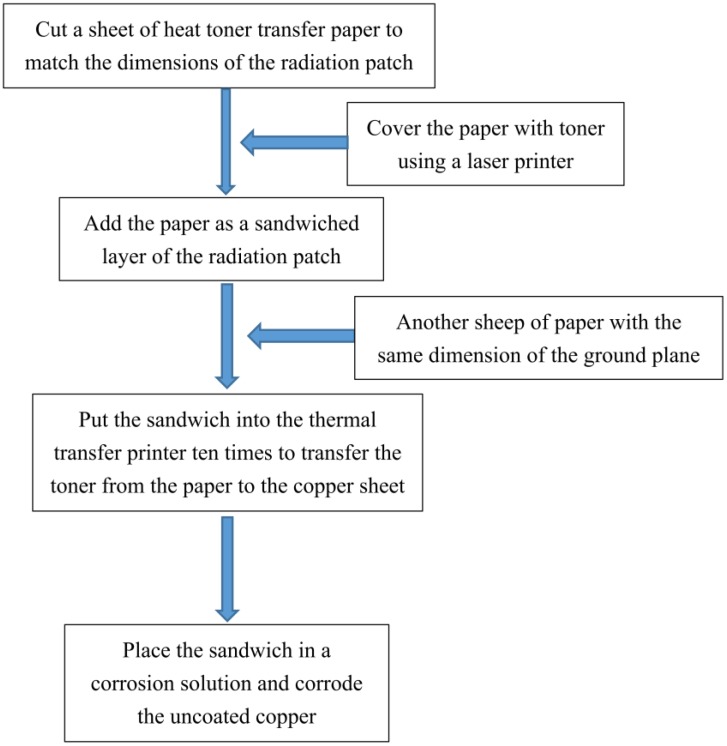
Flow chart of antenna production in laboratory.

**Figure 8:**
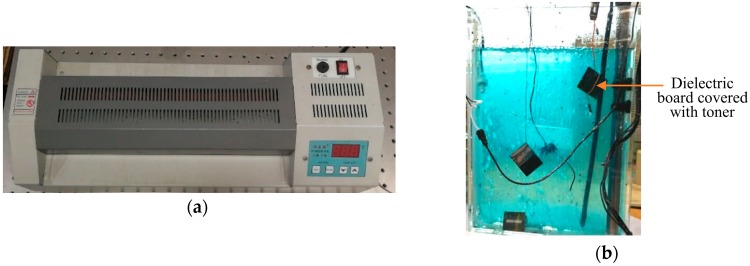
The equipment used to produce the patch antenna. (**a**) a thermal transfer printer; (**b**) corrosive liquid.

**Figure 9:**
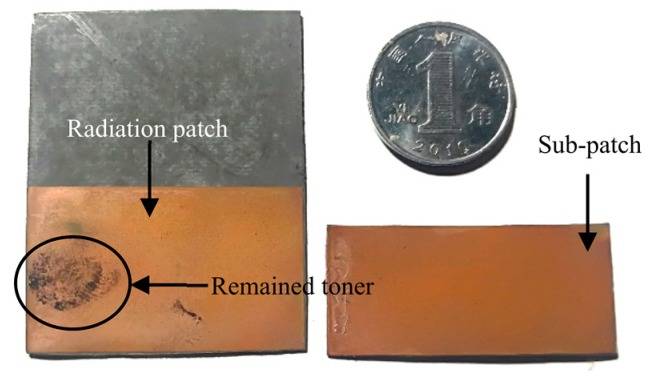
The manufactured crack sensor.

The flow chart seen in Figure 7 shows the equipment used to produce the patch antenna, as shown in Figure 8. In addition, the manufactured crack sensor is shown in Figure 9. The parameters of the sensor are the same as in the simulation section.

### 4.2. Instrumentation Setup

**Figure 10:**
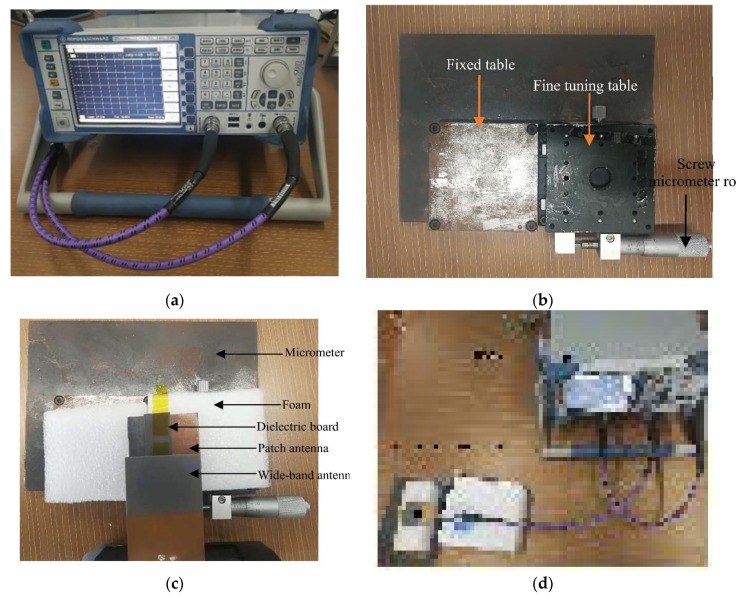
The experimental setup. (**a**) The network analyzer used in experiment; (**b**) The micrometer; (**c**) The testing sensor; (**d**) The micrometer with the crack sensor.

The testing device for crack sensing is established and shown in Figure 10. A micrometer shown in Figure 10c consists of a fixed table, a fine-tuning table and a screw type micrometer rod. The minimum step advance of the screw micrometer rod is 0.01 mm. The fine-tuning table can be pushed by the screw micrometer rod to move relative to the fixed table and then simulate an expanding crack. Two pieces of foam were attached to the fixed table and the fine-tuning table to isolate the crack sensor from the tables. This helped to minimize the electromagnetic interference caused by the crack simulator. The rectangular patch antenna was attached to the foam over the fixed table by glue, and the sub-patch was attached to the fine-tuning table. When the screw rod is rotated, a crack forms and then expands, which leads to the increase of the overlapped length, thereby changing the resonant frequency of the sensor. To simplify the testing device, the installed method for the experimental sensor is different from the way described at Section 2, and the moving direction between the sub-patch and the patch antenna is opposite compared with the moving direction described at Section 2.

A wide-band antenna is used to give a plane wave feeding to the sensor and, thus, to receive the backscattering of the patch antenna. To reduce the background reflection, the wide-band antenna was set about 2 cm or closer above the crack sensor. A vector network analyzer (VNA) analyzed the signal of the wide band antenna received from the crack sensor. The sweeping range of the VNA was chosen to range from 2 GHz to 3 GHz, and the working band of the wide-band antenna’s range is from 1 GHz to 2.8 GHz to match the crack sensor and the VNA. The experiment was carried out with the overlapped length changing from 1.5 mm to 3 mm; thus, the linearity suffered when the overlapped length was below 1.5 mm. By receiving and transmitting the backscattering signal, the resonant frequency can be wirelessly analyzed. 

The experiment was carried out as follows: First, the sub-patch was set over the patch antenna with the overlap length at 1.5 mm. Then the micrometer moved with each incremental step set at 0.1 mm, thereby forming an expanded crack between two pieces of foam. The signal from the backscattering was then monitored by a VNA to get the radar cross section (RCS) curve of the sensor for each step. By extracting the resonant frequency at the local minimum of each curve, the shift of the resonant frequency was obtained and is described in Section 4.3.

### 4.3. Results and Discussion

**Figure 11:**
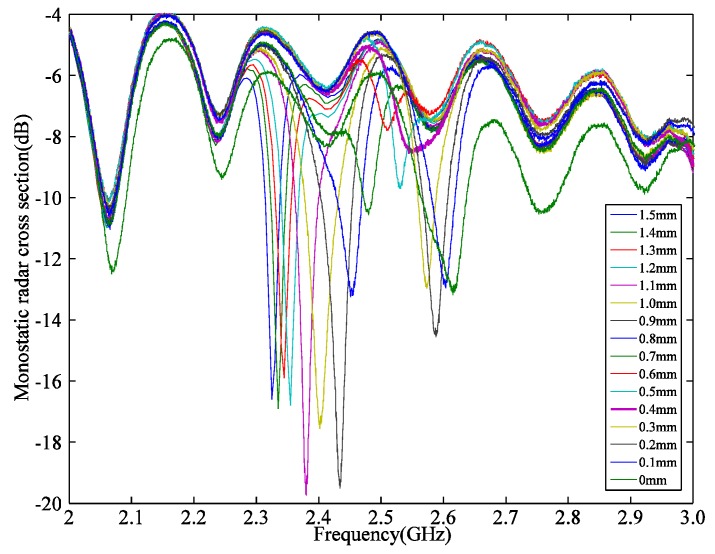
The monostatic radar cross section (RCS) in the experiment.

**Figure 12:**
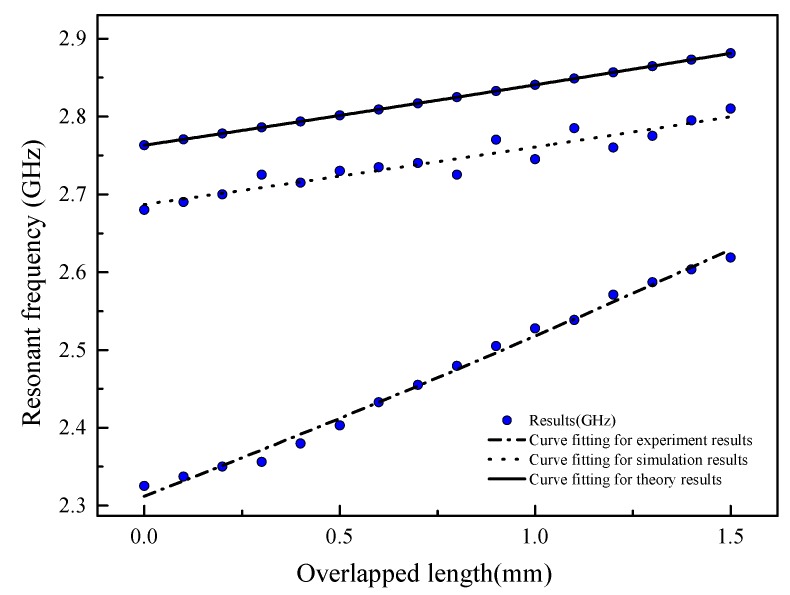
The relationship between resonant frequency and overlapped length in theory calculation, numerical simulation, and in the experiment.

Based on the experimental results, Figure 11 shows the radar cross section curve. Figure 12 shows the relationship between the fundamental resonant frequency and overlap length in the calculated theory of Equation (10). Table 3 compares the results of sensitivity in GHz, the measuring range in GHz, and correlation coefficient of the fitted line as they pertain to theory calculation, numerical simulation and the experiment. The correlation coefficient of the fitted line of each group are all over 0.9. The sensitivity in theory calculation is close to the numerical simulation results, but the sensitivity measurement in the experiment is more than double that of the theoretical and numerical results. The sensitivity difference could be due to the air gap between the sub-patch and the patch antenna. Moreover, the measuring range of each method varied slightly. This discrepancy is probably due to the following reasons:

(1). The impact of the substrate of the sub-patch was ignored in theoretical calculation. However, the impact of the substrate was taken into consideration in both the numerical simulation and the experiment.

(2). The patch antenna produced in the laboratory became defective when a little toner remained on the surface of the radiation patch, as shown in Figure 9.

(3). The monostatic RCS is measured in near field, so the value of the monostatic RCS during the frequency sweep would be affected by the interrogating distance, which would decrease the treatability and accuracy of the measuring results.

(4). The use of a micrometer can produce electromagnetic interference, which was not a consideration in either the theoretical calculation or numerical simulation. 

## 5. Conclusions

This paper introduces a novel crack sensor based on a patch antenna with an overlapping sub-patch. Using the theory of cavity model, the authors revealed a linear relationship between the fundamental resonant frequency in both the longitudinal direction and the overlapped length of the crack sensor. One prototype was modeled and fabricated for numerical simulation and experiments. The resonant frequency of the crack sensor was wirelessly interrogated by a wide band antenna, and the results showed a sensitivity of 120.24 MHz/mm on average within an effective measuring range of 1.5 mm. 

## Figures and Tables

**Table 1:** Setting domain for parameters of the crack sensor.

Parameters	W **(mm)**	$L_{r}\;(\mathrm{mm})$	$L_{s}\;(\mathrm{mm})$	$L_{c}\;(\mathrm{mm})$	$L_{t}\;(\mathrm{mm})$	f (GHz)	$\varepsilon_{e}$	k	m	n
Dimensions	35	18–22	13–18	<40	<44	2.4	2.2	1	1	0

**Table 2:** Final dimensions of the crack sensor.

Parameters	W **(mm)**	$L_{r}\;(\mathrm{mm})$	$L_{o}\;(\mathrm{mm})$	$L_{e}\;(\mathrm{mm})$	$L_{t}\;(\mathrm{mm})$	f (GHz)	$\varepsilon_{e}$	k	m	n
Dimensions	35	20.6	16	<36.6	43.4	2.4	2.2	1	1	0

**Table 3:** The sensitivity, measuring range and correlation coefficient of the fitted line in theory calculation, numerical simulation, and the experiment.

Serial	Sensitivity (MHz/mm)	Measuring Range (GHz)	Correlation Coefficient of the Fitted Line
Theory calculation	78.72	2.793–2.881	0.9999
Numerical simulation	86.67	2.68–2.81	0.9048
Experiment	195.33	2.326–2.619	0.9919

## References

[1] Leong W.H., Staszewski W.J., Lee B.C., Scarpa F. (2005). Structural health monitoring using scanning laser vibrometry: III. Lamb waves for fatigue crack detection. Smart Mater. Struct..

[2] Torres M.A., Ruiz S.E. (2007). Structural reliability evaluation considering capacity degradation over time. Eng. Struct..

[3] Johnson H.H., Morlet J.G., Troiano A.R. (1958). Hydrogen, crack initiation, and delayed failure in steel. Trans. Met. Soc. AIME.

[4] Guo H., Xiao G., Mrad N., Yao J. (2011). Fiber optic sensors for structural health monitoring of air platforms. Sensors.

[5] Tsuda H., Lee J., Guan Y. (2006). Fatigue crack propagation monitoring of stainless steel using fiber Bragg grating ultrasound sensors. Smart Mater. Struct..

[6] Kong X., Li J., Collins W., Bennett C., Laflamme S., Jo H. (2017). A large-area strain sensing technology for monitoring fatigue cracks in steel bridges. Smart Mater. Struct..

[7] Smyl D., Liu D. (2019). Damage tomography as a state estimation problem: crack detection using conductive area sensors. IEEE Sens..

[8] Karhunen K., Seppänen A., Lehikoinen A., Blunt J., Kaipio J.P., Monteiro P.J. (2010). Electrical Resistance Tomography for Assessment of Cracks in Concrete. Aci. Mater. J..

[9] Usamentiaga R., Venegas P., Guerediaga J., Vega L., Molleda J., Bulnes F.G. (2014). Infrared Thermography for Temperature Measurement and Non-Destructive Testing. Sensors.

[10] Radzieński M., Kudela P., Marzani A., De Marchi L., Ostachowicz W. (2019). Damage Identification in Various Types of Composite Plates Using Guided Waves Excited by a Piezoelectric Transducer and Measured by a Laser Vibrometer. Sensors.

[11] Lee C., Zhang A., Yu B., Park S. (2017). Comparison Study between RMS and Edge Detection Image Processing Algorithms for a Pulsed Laser UWPI (Ultrasonic Wave Propagation Imaging)-Based NDT Technique. Sensors.

[12] Caizzone S., DiGiampaolo E., Marrocco G. (2014). Wireless crack monitoring by stationary phase measurements from coupled RFID tags. IEEE Trans. Antennas Propag..

[13] Yi X., Cho C., Cooper J., Wang Y., Tentzeris M.M., Leon R.T. (2013). Passive wireless antenna sensor for strain and crack sensing—Electromagnetic modeling, simulation, and testing. Smart Mater. Struct..

[14] Raghunathan V., Kansal A., Hsu J., Friedman J., Srivastava M. Design considerations for solar energy harvesting wireless embedded systems. Proceedings of the 4th International Symposium on Information Processing in Sensor Networks.

[15] Vullers R.J., Van Schaijk R., Visser H.J., Penders J., Van Hoof C. (2010). Energy harvesting for autonomous wireless sensor networks. IEEE Solid-State Circuits Mag..

[16] Wang L., Yuan F.G. Energy harvesting by magnetostrictive material (MsM) for powering wireless sensors in SHM. Proceedings of the SPIE Smart Structures and Materials + Nondestructive Evaluation and Health Monitoring.

[17] Tchafa F.M., Huang H. (2019). Microstrip patch antenna for simultaneous temperature sensing and superstrate characterization. Smart Mater. Struct..

[18] Huang Y., Chen Y., Wu T. (2010). A passive wireless hydrogen surface acoustic wave sensor based on Pt-coated ZnO nanorods. Nanotechnology.

[19] Butler J.C., Vigliotti A.J., Verdi F.W., Walsh S.M. (2002). Wireless, passive, resonant-circuit, inductively coupled, inductive strain sensor. Sens. Actuator A-Phys..

[20] Yi X., Wu T., Wang Y., Leon R.T., Tentzeris M.M., Lantz G. (2011). Passive wireless smart-skin sensor using RFID-based folded patch antennas. Int. J. Smart Nano Mater..

[21] Zaid J., Abdulhadi A.E., Denidni T.A. (2019). Miniaturized Multi-Port Microstrip Patch Antenna Using Metamaterial for Passive UHF RFID-Tag Sensor Applications. Sensors.

[22] Borgese M., Dicandia F.A., Costa F., Genovesi S., Manara G. (2017). An inkjet printed chipless RFID sensor for wireless humidity monitoring. IEEE Sens. J..

[23] Girbau D., Ramos A., Lazaro A., Rima S., Villarino R. (2012). Passive Wireless Temperature Sensor Based on Time-Coded UWB Chipless RFID Tags. IEEE T Microw Theory.

[24] Tchafa F.M., Huang H. (2018). Microstrip patch antenna for simultaneous strain and temperature sensing. Smart Mater. Struct..

[25] Donelli M. (2018). An RFID-Based Sensor for Masonry Crack Monitoring. Sensors.

[26] Lange D.A. Passive Wireless Sensors for Monitoring Behavior of Recycled Aggregate Concrete. https://rosap.ntl.bts.gov/view/dot/36891.

[27] Mohammad I., Gowda V., Zhai H., Huang H. (2011). Detecting crack orientation using patch antenna sensors. Meas. Sci. Technol..

[28] Mohammad I., Huang H. (2010). Monitoring fatigue crack growth and opening using antenna sensors. Smart Mater. Struct..

[29] Marindra A.M.J., Tian G.Y. (2018). Chipless RFID sensor tag for metal crack detection and characterization. IEEE T Microw Theory.

[30] Xue S., Xu K., Xie L., Wan G. (2019). Crack sensor based on patch antenna fed by capacitive microstrip lines. Smart Mater. Struct..

[31] Caizzone S., DiGiampaolo E. (2015). Wireless Passive RFID Crack Width Sensor for Structural Health Monitoring. IEEE Sens. J..

[32] Xue S., Yi Z., Xie L., Wan G., Ding T. (2019). A Displacement Sensor Based on a Normal Mode Helical Antenna. Sensors.

[33] Balanis C.A. (2016). Antenna Theory: Analysis and Design.

